# Synthetic biology for combating leishmaniasis

**DOI:** 10.3389/fmicb.2024.1338749

**Published:** 2024-02-01

**Authors:** Shweta Khandibharad, Shailza Singh

**Affiliations:** Systems Medicine Laboratory, National Centre for Cell Science, Pune, India

**Keywords:** synthetic biology, leishmaniasis, therapeutics, diagnostics, vaccine

## Abstract

Leishmaniasis is a neglected tropical disease caused by protozoan parasites of the *Leishmania* genus. Despite the efforts to control and treat the disease, it still remains a major public health problem in many countries. Synthetic biology is a rapidly evolving interdisciplinary field that combines biology, engineering, and computer science to design and construct novel biological systems. In recent years, synthetic biology approaches have shown great promise for developing new and effective strategies to combat leishmaniasis. In this perspective, we summarize the recent advances in the use of synthetic biology for the development of vaccines, diagnostic tools, and novel therapeutics for leishmaniasis.

## Introduction

Leishmaniasis, a parasitic disease caused by the protozoan parasite of the *Leishmania* (L.) genus, primarily spreads to humans through the bites of sand flies belonging to the *Phlebotomus* and *Lutzomyia* genera. Endemic in regions such as Asia, Africa, the Americas, and the Mediterranean, this disease sees an annual global incidence of 1.5 to 2 million new cases, putting 350 million individuals at risk and resulting in approximately 70,000 fatalities annually (Torres-Guerrero et al., 2017). The main clinical forms include Cutaneous Leishmaniasis (CL) from *Leishmania major*, Visceral Leishmaniasis (VL) from *Leishmania donovani* and *L. infantum*, and Mucocutaneous Leishmaniasis (MCL) from *Leishmania braziliensis*. Current estimates place CL incidence between 700,000 and 1.2 million cases annually, with over 95% occurring in the Americas, the Mediterranean basin, the Middle East, and Central Asia. Brazil, China, Ethiopia, India, Kenya, Nepal, Somalia, and Sudan contribute to more than 95% of reported VL cases to the World Health Organization (WHO), marking a significant decline from previous estimates of 400,000 cases per year. Risk factors for leishmaniasis include poverty, population mobility, malnutrition, poor hygiene, and immunocompromised states (Burza et al., 2018; Mann et al., 2021).

Leishmaniasis can present in various forms, including self-healing skin lesions confined to the transmission site (CL), lesions spreading to mucosal areas (MCL), or a potentially fatal systemic disease affecting major organs like the liver (kala azar or VL). Additionally, individuals treated for VL often develop post-kala-azar dermal leishmaniasis (PKDL), a persistent skin condition that can sustain VL transmission in communities (Ashwin et al., 2021). The risk of co-infection significantly influences disease progression and severity. Examining reported cases globally, countries with malaria, VL, and CL reveal overlapping instances in the Americas (Central and South), Africa, and Asia, indicating that at least 38 countries face a risk of co-infection, as supported by seminal data. In India, 5.9% of patients with splenomegaly and fever are diagnosed with both malaria and leishmaniasis (Ornellas-Garcia et al., 2023). Notably, the most extensively studied co-infection involves *Leishmania* spp. and the human immunodeficiency virus (HIV), as the presence of one alters the natural course of the other. In the context of *L. major* which is confined to the skin, HIV increases the risk of severe and widespread Tegumentary leishmaniasis (TL), with some HIV-infected individuals developing visceral leishmaniasis (Martínez et al., 2018).

### Current therapeutic options for the treatment of leishmaniasis through pharmaceutical agents

**Figure 1:**
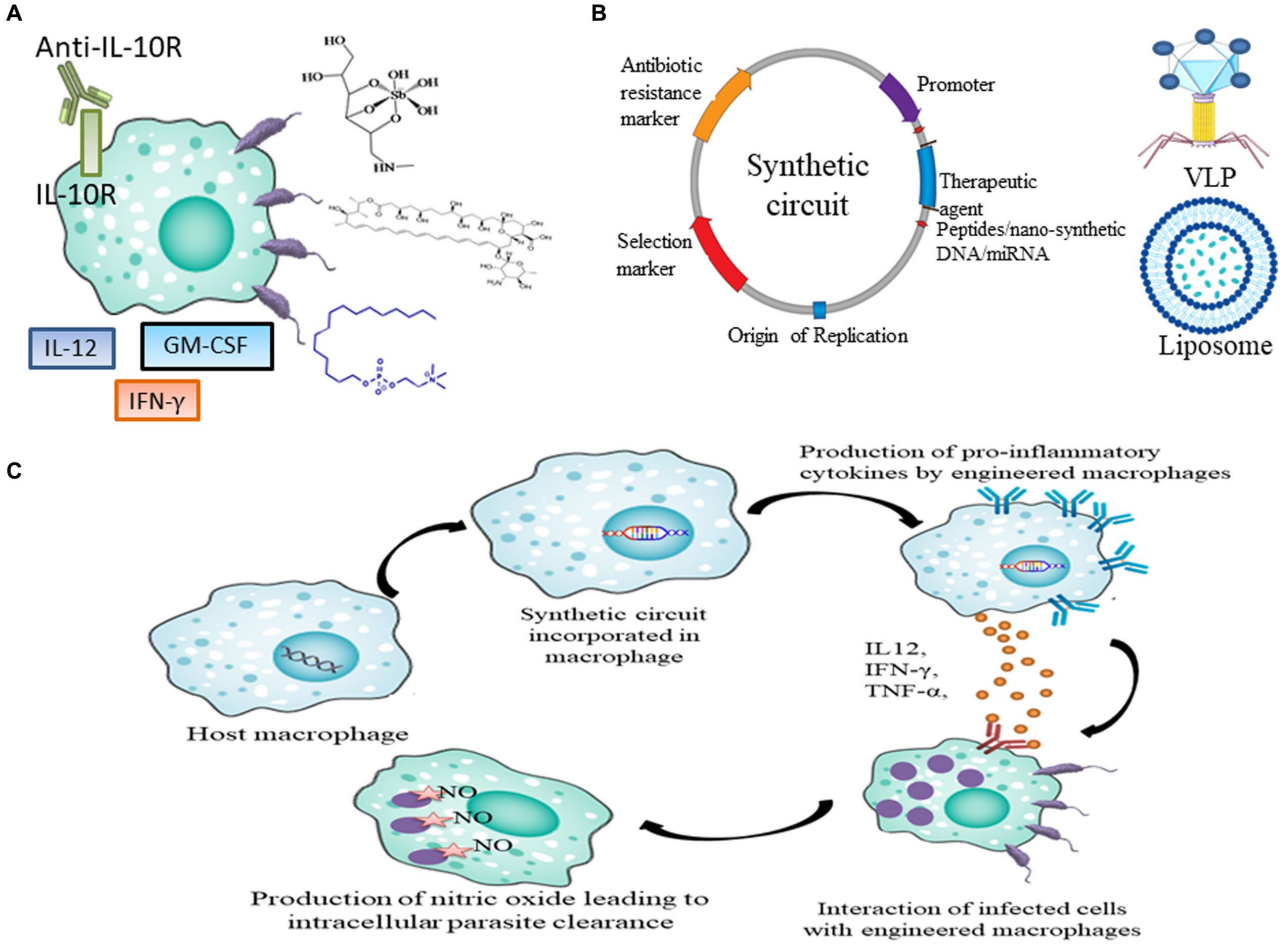
Current and future prospects of therapeutics in leishmaniasis. **(A)** Current drugs and immunotherapy available for treating leishmaniasis which includes SSG, AmB, Miltefosine, IL-12, IFN-γ, GM-CSF and anti-IL10R antibody. **(B)** Genetic parts of synthetic circuit and its delivery in macrophages through VLPs and Liposome **(C)** Pipeline to express therapeutic agents like peptides, nano-synthetic DNA and miRNA in macrophages to achieve disease resolving effect.

The existing treatments for leishmaniasis are constrained and come with notable limitations. Essential criteria for drug or vaccine administration include cost-effectiveness, safety, long-term efficacy, and the ability to reduce infection transmission. Sodium stibogluconate (SSG), a pentavalent antimony Sb(V), has been a frontline agent in anti-leishmanial chemotherapy. The first therapeutic model is predicated on a prodrug concept, involving the reduction of Sb(V) to trivalent antimony Sb(III) (Figure 1A). Modulation of immune response in CL patients treated with SSG was observed which included a decline in antigen-specific CD4+ T cell proliferation along with CD8+ T cell depletion. Furthermore, the state of recovery or healing is distinguished by a diminished presence of circulating regulatory T cells, decreased Interferon-gamma (IFN-γ) production, and a general reduction in polyfunctional CD4+ T cells (Lakhal-Naouar et al., 2015).

In the second model, Sb(V) exhibits anti-leishmanial properties by forming complexes with ribose-containing molecules, thereby inhibiting type I DNA topoisomerases. Despite its prominence, the efficacy of SSG is curtailed by escalating resistance (Ghorbani and Farhoudi, 2018; Brindha et al., 2021). Antimony resistance is further associated with ATP-binding cassette (ABC) family transporters, multidrug-resistance protein A (MRPA), and pentamidine-resistant protein 1 (PRP1), which function as efflux pumps for antimonials (Singh et al., 2012).

Amphotericin B (AmB) is a polyene antifungal medication widely employed to combat systemic fungal infections (Figure 1A). It exhibits strong affinity toward ergosterol, the principal sterol found in fungal and leishmanial cell membranes (Singh et al., 2012). Despite the rarity of reports on AmB resistance, it is a critical concern, especially given its use in cases of leishmaniasis relapse (Alpizar-Sosa et al., 2022).

For the treatment of VL and CL, a prompt and safe short-course treatment involving liposomal amphotericin B (L-AmB) is commonly administered as an alternative to AmB. A single-dose regimen of L-AmB has proven to be highly effective, secure, and potentially less burdensome (Mondal et al., 2014). However, they have been reported to be less effective against CL treatment (Daftarian et al., 2013), instances of relapse in some patients, and the treatment is associated with higher costs (Özsoylu, 2003).

As the sole oral treatment for leishmaniasis, miltefosine facilitates outpatient care, enabling the expansion of therapeutic interventions (Figure 1A; Van Henten et al., 2021). This medication acts by hampering phosphatidylcholine production and inhibiting cytochrome c oxidase (Pinto-Martinez et al., 2018). Functioning as an immunomodulator, miltefosine reinstates sensitivity of parasite to IFN-γ, enhances the early production of Interleukin-12 (IL-12) and Tumor necrosis factor- alpha (TNF-α), regulates Th1/Th2 responses by promoting proliferation of CD4+ and CD8+ T cells, and augments Reactive oxygen species (ROS) production (Neira et al., 2023). CL induced by *L. braziliensis* in Bolivian patients exhibited an 88% rate of successful cure (Machado et al., 2010). Common side effects encompass gastrointestinal disturbances (Van Henten et al., 2021), and it has been documented to possess teratogenic properties as well (Ware et al., 2021).

Different cytokines, including GM-CSF (granulocyte-macrophage colony-stimulating factor), IL-12 and IFN-γ have been employed in both monotherapy and combinatorial immunotherapy treatment (Figure 1A). Monoclonal antibody against Interleukin-10 (IL-10) receptor also has been used to induce antiparasitic activity via activation of Nitric oxide (NO). It was observed that GM-CSF in combination with meglumine or a combination of *L. major* antigens, such as LmST1 + LeIf6 + HSP83, is effective in treating CL (Sasidharan and Saudagar, 2021). When *L. donovani*-infected mice were treated with a single dose of anti-IL-10R monoclonal antibody, Sb(V) and amphotericin individually, the percentage of parasites killed in the liver increased to approximately 63, 72, and 76%, respectively (Figure 1A; Sasidharan and Saudagar, 2021). One major barrier preventing the development and use of cytokine immunotherapy as therapeutic and vaccine is the unification of innate and acquired immunity together with the unavailability of information on the human immune response (Yadagiri et al., 2023).

Local treatment modalities like photodynamic therapy, cryotherapy, and thermotherapy are employed in the treatment of leishmaniasis. Research is ongoing into various vaccine approaches, encompassing those utilizing whole-killed parasites, fractionated *Leishmania* antigens, live-attenuated pathogens, and recombinant proteins produced through genetic modification (Pradhan et al., 2022). The applications of treatment measures are enlisted in Table 1.

**Table 1:** Current treatment options for leishmaniasis.

Drugs and therapy	Type of leishmaniasis	Mode of action	Limitations and side effects	References
Pentavalent antimony Sb(V)- Sodium stibogluconate and meglumine antimoniate	VL and CL	Can affect enzymes in the liver alanine aminotransferase (ALT) and aspartate aminotransferase (AST).	Resistance, cardiac and renal toxicity	Ghorbani and Farhoudi (2018)
Amphotericin B	MCL, VL and HIV–*Leishmania* coinfection	Binds to ergosterol of parasite and cause membrane leakage	Fever, chills, and thrombophlebitis, hypokalemia, nephrotoxicity, myocarditis	Sundar and Chakravarty (2010) and Shirzadi (2019)
Liposomal Amphotericin B (Ambisome)	VL and CL	Binds to ergosterol of parasite and cause membrane leakage	Unstable at temperature exceeding 25°C	Frézard et al. (2022)
Miltefosine	VL and CL	Interfere with the leishmanial membrane lipids and mitochondrial function	Gastrointestinal upset and teratogen	van Henten et al. (2021) and Ware et al. (2021)
Photodynamic therapy	VL and CL	Production of Reactive oxygen species (ROS) through photosensitizer	Toxicity, high cost, and difficult administration	Vital-Fujii and Baptista (2021)
Cryotherapy	CL	Lesions are treated with liquid Nitrogen or dry ice	Ambiguity in results, high cost and maintenance	Shaddel et al. (2018)
Thermotherapy	CL	Delivery of external heat to lesion	Painful	Kämink et al. (2021)
Whole killed parasite Vaccines	CL	Prophylactic and therapeutic vaccines	Poorly defined and variable in potency	Kedzierski (2010)
Fractionated *Leishmania* vaccines	Canine VL and VL	LAg, induces protective immunity	Production, standardization and purification	Srivastava et al. (2016)
Live-attenuated pathogens	VL and CL	Differential protection	Toxicity	Volpedo et al. (2022) and Pandey et al. (2020)
IFN-γ therapy	VL	Decreases IL10 production	Dosage, non-sustainable IFN-g production	Kumar et al. (2020) and Kima and Soong (2013)

### Drug resistance and identification of novel targets in leishmaniasis

Drug resistance in leishmaniasis refers to the development of molecular resistance mechanisms in a population of previously sensitive *Leishmania* parasites, which results in a decline or absence of activity of the particular agent against that population (Wijnant et al., 2022). Some of the potential drug targets in *Leishmania* parasite are, ergoterol, enzymes of glycolytic pathway, DNA topoisomerase, polyamines, redox metabolism inducing enzymes, dihydrofolate reductase and Mitogen-activated protein kinase (MAPK) (Raj et al., 2020). Traditionally, resistance is caused by genetic mutations that decrease the parasite’s reaction to a drug when it is under pharmacological pressure as parasite’s genome is highly plastic (Ponte-Sucre et al., 2017). At present, the most discerning and sensitive method is bulk genome sequencing (BGS). Novel DR-associated genomic alterations in the nuclear and kinetoplast genomes can be found attributable to this genotyping method (Domagalska et al., 2023).

Novel antileishmanial targets and resistance genes against five important clinically relevant antileishmanial drugs—Sb(III), miltefosine, paromomycin, AmB, and pentamidine—were identified by cosmid sequencing of the *L. infantum* genome library. Genes P299 and ARM58 may be novel targets that induce Sb(III) resistance, *L. infantum* genes, LinJ.29b (LinJ.29.2250) and LinJ.30 (LinJ.30.2270) associated with ergosterol synthesis and phospholipid translocation may induce miltefosine resistance; LinJ.26.2620 has been proposed to interact with AmB and induce resistance against it; and LinJ.06b, whose function is unknown but has been reported to elucidate pentamidine and paromycine resistance (Gazanion et al., 2016).

**Figure 2:**
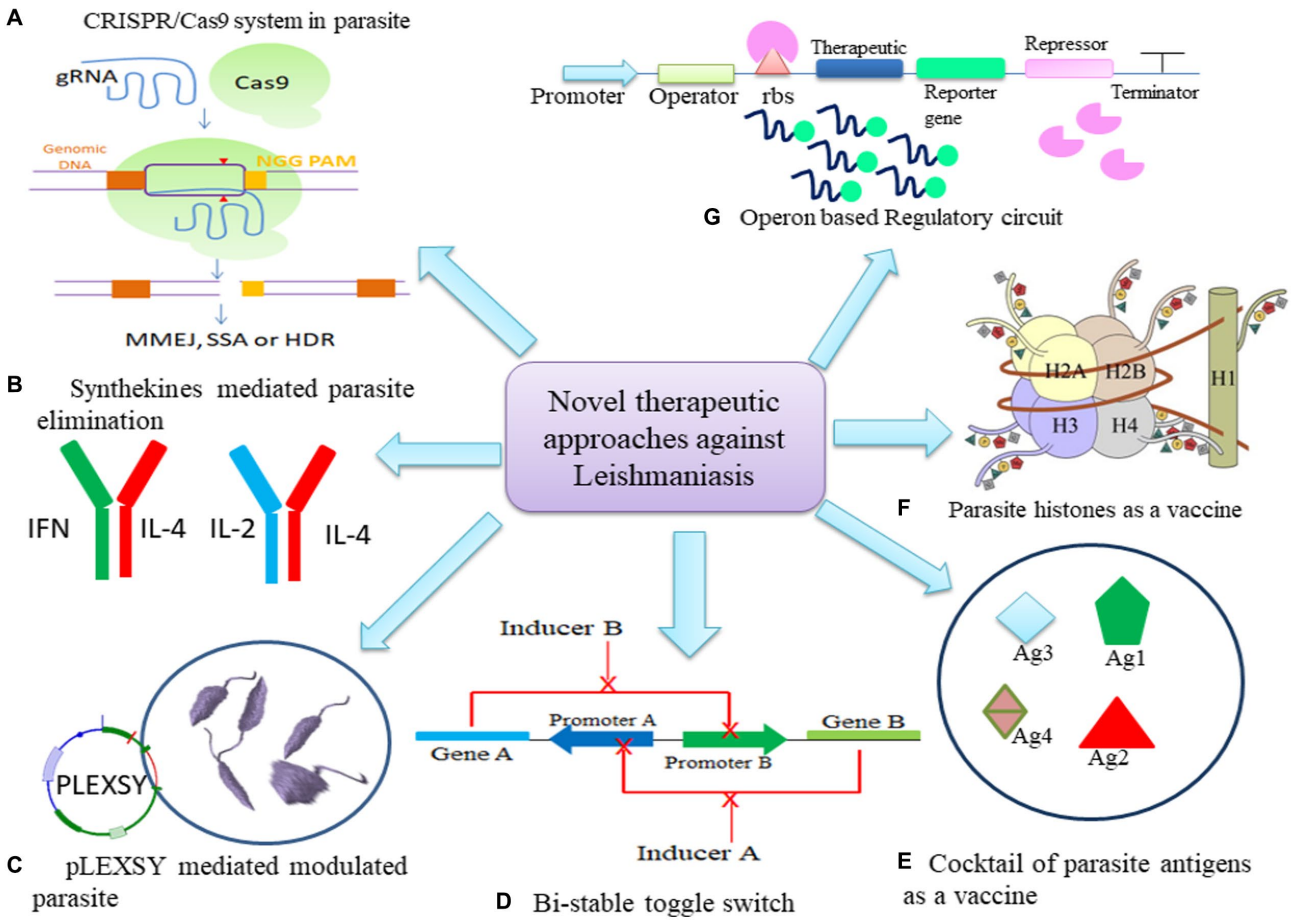
Potential of Synthetic biology tools as therapeutics in leishmaniasis. **(A)** CRISPR/Cas9 mechanism to mutate parasite. **(B)** Engineering synthekines to regulate host immune response to eliminate parasite. **(C)** Modulation of parasite through pLEXSY system **(D)** Bi-stable toggle switch as multi-therapeutic approach to combat resistance in parasite **(E)** Cocktail of parasite antigens can be cloned to modulate host macrophage response. **(F)** Histones of parasite can be engineered in synthetic circuit to develop stronger immune response. **(G)** Operon may be modulated to express at spatio-temporal level to express therapeutic.

The Clustered regularly interspaced short palindromic repeats and CRISPR-associated protein 9 (CRISPR/Cas9) system is being regarded as a prospective strategy for generation of efficacious vaccines targeting leishmaniasis (Figure 2A; Salehi Sangani et al., 2019). LeishGEdit provides templates for the transcription of single guide RNA (sgRNA) in cells expressing both Cas9 and T7 RNA polymerase, editing cassettes selectable by drugs that utilize a modular array of plasmids as templates. The pT plasmids may serve as a vector backbone to clone CRISPR/Cas9 system facilitate the amplification of genes conferring drug resistance for knockout purposes (Beneke and Gluenz, 2019). Using a synthetic guide RNA 30 bp upstream and downstream regions of leucine-rich repeats (LRR), multiple genes in *L. infantum* were flanked to validate resistance marker of Sb(III) and Puromycine. Generation of these knockouts highlighted that LRR genes may confer a dual advantage to *Leishmania*, potentially contributing to drug resistance in both promastigotes and amastigotes, along with an enhanced ability for adaptation to macrophage infections (Fernandez-Prada et al., 2018).

CRISPR/Cas9 system can be introduced in *L. donovani* to develop vectors expressing gRNA under *L. donovani* rRNA promoter and ribozyme derived from hepatitis delta virus. To repair DNA double strand breaks in genome, *L. donovani* adopted homology directed repair, microhomology-mediated end-joining and single strand annealing methods (Zhang and Matlashewski, 2015). Same group developed vector which expressed gRNA as well as Cas9 and its efficacy was evaluated in *L. donovani*, *L. major*, and *L. mexicana*. The group adopted co-targetting stratergy to generate mutants of A2 gene family and miltefosine transporter gene (Zhang et al., 2017). The group also developed a novel constitutive *Staphylococcus aureus* Cas9 vector for efficient expression of targeted gene therapy in *L. donovani*, *L. major*, and *L. mexicana* (Zhang and Matlashewski, 2019). Mutants lacking arginine transporter (AAP3.2) were generated using CRISPR/Cas9 which prevents *Leishmania* promastigotes to upregulate AAP3 expression upon arginine deprivation which made them incapable to proliferate in the livers of BALB/c mice or in THP-1 macrophages. This study demonstrated that the development of intracellular parasites depends on the ability to sense host nutrients (Goldman-Pinkovich et al., 2020). CRISPR/Cas9 vector was constructed with Dihydrofolate Reductase-Thymidylate Synthase (DHFR-TS) promoter which regulated the expression of the Cas9 endonuclease while gRNA was expressed under the control of the U6snRNA promoter and terminator to knockout paraflagellar rod-2 region in *Leishmania* parasite (Sollelis et al., 2015). In order to create mutant phenotypes in *Leishmania* and other kinetoplastids, stable Cas9 was expressed in *L. mexicana*, *L. major* and *T. brucei*. gRNA expression for lipophosphoglycan (LPG) production and variant surface glycoprotein (VSG) was achieved *in vivo* to generate *Leishmania* mutants with defective flagellar motility (Beneke and Gluenz, 2019). CRISPR/Cas9 encoding vector pLdCN can be delivered in *Leishmania* cells using transfection employing electrophoresis stratergy in Tb-BSF buffer (Zhang et al., 2020).

### Synthetic biology and its potential application in cytokine modulation in leishmaniasis

Synthetic biology aims to enhance immunotherapeutics, exemplified by the integration of synthetic biology and genetic engineering. Novel tools such as synthetic cytokines, cytokine receptors, and constitutively active cytokine receptor variants are being developed to enhance and fine-tune immunotherapeutic strategies (Scheller et al., 2019). Anomalous activation of cytokine signaling, often stemming from excessive cytokine production or hyperactive mutated receptors, is a common precursor to severe diseases, including those of a life-threatening nature such as chronic inflammatory conditions (Fajgenbaum and June, 2020).

Combining two dominant negative (DN) cytokine variants, each binding to only one receptor subunit, gives rise to synthekines (Zheng et al., 2022; Figure 2B). These involve cytokines like Interleukin-4 (IL-4), Interleukin-2 (IL-2), and IFNω. Specifically, synthekines such as IFNDN-IL-4DN and IL-2DN-IL-4DN activate nonnatural receptor pairs, IFNAR2-IL-4Rα and IL-2Rβ-IL-4Rα. Human T cells cultured with either natural ligands or engineered synthekines exhibit *in vitro* activation of distinct transcription factors and, in some cases, entirely different signaling pathways (Scheller et al., 2019). Engelowski et al. (2018) made on/off switchable high-affinity GFP- and mCherry-nanobodies which were linked to the transmembrane and intracellular domains of IL-6/IL-11 and IL-23 cytokine receptors, namely gp130 and IL-12Rβ1/IL-23R. Through homo- and heterodimeric GFP:mCherry fusion proteins serving as synthetic cytokine-like ligands, canonical signaling was effectively induced both *in vitro* and *in vivo*. The utilization of Synthetic cytokine receptors (SyCyR) ligands demonstrated that Interleukin-23 (IL-23) receptor homodimerization triggers activation and signal transduction resembling IL-23. Additionally, trimeric receptor assembly fosters trans-phosphorylation among cytokine receptors accompanied by associated Janus kinases and STAT1/3. The potential application of this system can be done in transgenic mice for cell-type specific response.

A synthetic cytokine converter for mammalian cells was engineered to quantify levels of TNF and Interleukin-22 (IL-22) associated with psoriasis. This converter utilized serially linked receptor-based synthetic signaling cascades, employing AND-gate logic to process the levels of these proinflammatory cytokines. Subsequently, it triggers the expression of therapeutic levels of the anti-inflammatory/psoriatic cytokines IL-4 and IL-10, known for their immunomodulatory effects in patients. Microencapsulated cytokine converter transgenic designer cells, when implanted, demonstrated insensitivity to simulated bacterial and viral infections, as well as inflammation unrelated to psoriasis. These designer cells specifically averted the onset of psoriatic flares, alleviated acute psoriasis, improved psoriatic skin lesions, and restored normal skin-tissue morphology in mice (Schukur et al., 2015).

Leishmaniasis being an inflammatory disease, triggers the activation of immune cells, initiating diverse signaling cascades. The immune cells generate both pro-inflammatory and anti-inflammatory cytokines as a response to infection. An imbalance in the cellular homeostasis due to levels of these cytokines has the potential to alter the immune response paradigm, contributing to the progression of the disease. To reinstate an effective response for eliminating the parasite, synthetic biology can be harnessed to develop Immunotherapy strategies, centered on cytokine remodeling and reconstruction within immune cells, particularly sentinel cells such as macrophages, hold significant promise in this context.

### Applications of synthetic biology in leishmaniasis

Synthetic biology has been successfully augmented for devising synthetic circuits and its efficiency in *L. major* causing CL models were analyzed for achieving parasite eliminating effect. Synthetic circuit must have essential biological parts (Figure 1B) and it can be engineered in plasmid as vector backbone. The choice of therapeutics reportedly used can be cloned in plasmid. Further; the circuit can be delivered in immune cells via liposomal formulation, virus and nanoparticles (Figure 1B). Inside the immune cells synthetic circuit might either function as an episome or conjugate in genomic DNA to express the therapeutic to achieve disease resolving effect (Figure 1C).

Recent studies have demonstrated that *L. tarentolae*, a non-pathogenic species within the *Leishmania* genus, serves as a highly effective expression system. This species finds widespread application in gene manipulation, gene targeting, immunogenicity studies, investigations into gene functions, and the development of live vaccines. The generation of recombinant parasites through this system might facilitate the exploration of functional associations among diverse proteins and the assessment of anti-parasitic drug efficacy (Taheri et al., 2016). Vacas et al. (2017) devised pXG plasmid to express N- and C- terminal mCherry fusion protein in *Leishmania* spp. with hygromycine selection marker. *L. tarentolae* expression system (LEXSY) was employed to enhance the production of enhanced green fluorescent protein (EGFP) in various *Leishmania species*, including *L. tarentolae*, *L. major*, and *L. infantum*, through homologous recombination (Figure 2C). These parasites exhibited notable fluorescent signals both *in vitro* and allowed real-time visualization *in vivo*. The presence of viable *Leishmania species* in the amastigote form within adherent mouse macrophages, such as bone marrow-derived macrophages or the J774A.1 mice cell line, offers a more precise approach for evaluating the drug sensitivity profile of an anti-leishmanial compound (Bolhassani et al., 2011).

Mandlik et al., 2013 devised a robust bistable synthetic circuit (Figure 2D). The sphingolipid metabolism of the *Leishmania* parasite has made IPCS (inositol phosphoryl ceramide synthase) a desirable target. Ceramide choline phosphotransferase 4 was made specifically to increase the rate of phosphatidylcholine synthesis (SLS4 protein). A potential treatment for leishmaniasis would involve the site-specific delivery of the circuit into the parasite-infected macrophages. Further, *Leishmania* may control the activity of Protein kinase C (PKC) isoforms and nuclear factor-κB (NFκB) to ensure its safe intracellular survival. PKC isoforms are controlled by altering the regulatory domain’s or catalytic domain’s activity. It was observed that PKC- ζ was altered by increasing its affinity for its substrate when ceramide, which is concurrently generated during *Leishmania* infection, is present. The PB1 domain from PKC- ζ and the catalytic domain from PKC-α were combined to generate a chimeric PKC _ζα, which has the potential to rewire NFκB/RelA by phosphorylating IκB kinase (IKKβ), which in turn phosphorylates inhibitor of nuclear factor kappa B (IκB) and frees RelA for nuclear translocation and gene expression regulation (Mol et al., 2018).

Synthetic-DNA nanotechnology might be used to treat leishmaniasis and other infectious diseases. L-AmB was developed using nanotechnology to demonstrate its potential. As proteins, RNA, and DNA are biocompatible materials, integrating nanotechnology to medicine is becoming more and more plausible. If these DNA nanostructures and peptides with biological epitopes for cell receptors can be combined, the result might be a signal for cell differentiation. Such co-assemblies under investigation may be able to direct macrophage differentiation for parasite clearance (Mol et al., 2014).

Soni and Singh (2021a) demonstrated that peptide-based immuno-regulatory circuits have been developed to regulate the function of Suppressor Of Cytokine Signaling 1 (SOCS1), which can restore pro-inflammatory cytokine expression during infection, using synthetic biology (Figure 1B). They basically looked at the potential of synthetic biology to address and rewire the immune response from Th2 to Th1 type during the early stage of leishmanial infection, which is controlled by the SOCS1 and Suppressor Of Cytokine Signaling 3 (SOCS3), immune axis.

Kosey and Singh (2017) investigated a novel molecular motor myosin XXI which may result in newer leishmaniasis treatment modalities. It is a revolutionary concept that opens up a whole new world of therapeutic possibilities to employ a nanocircuit made up of a connected bistable switch and repressilator to cure the disease.

Nimsarkar et al. (2020) reported the use of synthetic module into two distinct inlays. One plasmid would be expressing the target gene; the other plasmid would be expressing the miRNA gene and GFP reporter protein. Such artificial circuits for, Mothers against decapentaplegic homolog 7 (SMAD7) and miR-146a were created in order to inhibit Transforming growth factor-β (TGF-β) signaling which promotes parasite signaling.

Bejugam and Singh (2018) designed a riboswitch where eGFP was used in place of the RNA polymerase III subunit 1 gene in a putative Theophylline binding riboswitch cassette in an *in vitro* reporter assay. This resulted in an apparent downregulation of the reporter gene’s expression when the Theophylline binding riboswitch was present. This switch was essential as Theophylline is necessary for the parasite to survive both in its promastigote and amastigote forms.

### Diagnosis of leishmaniasis with potential of vaccines strategies against *Leishmania spp* infection through synthetic biology perspective

The precise and sensitive detection of leishmaniasis is crucial for early diagnosis and effective management. However, currently available diagnostic tools have shortcomings, including low sensitivity and specificity. Synthetic biology methodologies have been applied to create innovative diagnostic tools for leishmaniasis. Programmable synthetic constructs can be employed to construct nanostructures within living cells in a controlled manner, eliciting leishmanicidal effects. The application of synthetic-DNA nanotechnology is essential for addressing infectious diseases like leishmaniasis. Recent endeavors focus on assembling multiple adjuvant elements on a DNA nanostructure to enhance immunostimulation capacity, indicating the potential of DNA nanostructures as innovative platforms for vaccine development. A tetrahedral DNA nanostructure has been utilized as a scaffold to assemble a model antigen, streptavidin (STV), and a representative adjuvant, CpG oligo-deoxynucleotides (ODN), forming a synthetic vaccine complex. A comprehensive understanding of the parasite–host interaction at the systems level and discerning intricacies in the interaction network may facilitate the construction of synthetic devices (Mol et al., 2015).

Bacteriophages were reported to be engineered to identify and differentiate various *Leishmania* spp. by displaying their antigenic peptides for diagnostic purposes which may also be used as a vaccination candidate in *L. infantum* infection (Coelho et al., 2015). Demonstrating high sensitivity and specificity in preclinical studies, this approach holds the potential to evolve into a diagnostic tool for leishmaniasis. Another avenue involves using synthetic biology to design biosensors capable of detecting leishmanial antigens or antibodies in patient samples. Biosensors are devices that integrate biological components with electronic or optical transducers to identify and quantify analytes (Martins et al., 2020). Biosensors rooted in synthetic biology have the potential to enhance the sensitivity and specificity of leishmaniasis diagnostics. Additionally, the CRISPR-Cas12a system has applications in the molecular identification of *Leishmania* spp. Common multi-copy target assays, such as PCR for leishmaniasis molecular diagnostics, include the highly conserved 18S ribosomal RNA gene (18S rDNA) and a segment of kinetoplast DNA (kDNA) minicircles (Dueñas et al., 2022). Despite the establishment of various diagnostic techniques, the scope for detection of parasite by developing more sensitive techniques is crucial.

Vaccines represent the most efficacious means of preventing infectious diseases. However, the intricate biology of the leishmanial parasite posed challenges in developing effective vaccines for leishmaniasis. Synthetic biology methodologies present novel opportunities for the creation of innovative vaccines against leishmaniasis. One example involves the engineering of bacterial cells using synthetic biology to express leishmanial antigens and facilitate their delivery to host cells. Notably, successful outcomes were observed in studies utilizing HHD-II mice (HLA-A0201 transgenic mice with both human HLA-A0201 and mouse H2-Kb genes) and FVB/N-DR1 mice (transgenic mice incorporating the HLA-DR1 gene). In these studies, HLA-A2-restricted peptides derived from *L. mexicana* or *L. major* gp63 and HLA-DR1-restricted peptides from *L. major* gp63 were utilized, showcasing promising results in preclinical investigations (Figure 2E) and this approach holds potential for further development into a viable vaccine candidate (Yasmin et al., 2022).

The evolution of vaccines for leishmaniasis spans three generations. The first generation involves the use of heat-killed and live attenuated forms of parasites. The second generation employs synthetic delivery agents expressing *Leishmania* antigens in bacteria or virus-like particles (VLPs) (Figure 1B). VLPs are self-assembling structures that mimic viruses in structure and function but lack infectious properties. Notable antigens in this generation include gp63, *Leishmania* Homolog for Receptors of Activated C Kinase (LACK), Kinetoplastid Membrane Protein-11 (KMP-11), Fucose Mannose Ligand (FML), and Monophosphoryl Lipid A (MPL-A) (Silva et al., 2020). Effective spatio-temporal expression of these proteins may induce long-term memory, serving as a preventive therapy.

The third generation involves DNA vaccines, which have been explored for VL. *Leishmania* histones have proven to be potent immunogens, eliciting a robust Th1 response in a murine model of *L. major* infection. Plasmids encoding *L. infantum* nucleosomal histones (H2A, H2B, H3, H4) were assessed in this context, demonstrating a strong Th1 response against CL (Figure 2F; Carrión, 2011; Moafi et al., 2019). Immuno-modulation of macrophages to polarize to M1 phenotype and to induce CD4+ T cells to polarize to Th1 phenotype was demonstrated by using *L. tarentolae* as a live vaccine against host as the parasite is reported to possess features that may be needed in an anti-*Leishmania* vaccine (Bandi et al., 2023). PpSP15 and PsSP9 were proposed to be a protective vaccine for *L. major* and *L. tropica* infection, however, T2A (a peptide that self-cleaves derived from a virus) linked PpSP15 and PsSP9 demonstrated to induce Th1 protective immunity in *L. tarentolae* infection model (Lajevardi et al., 2022).

Therefore, expressing parasite antigens on a non-infectious, stable vector or utilizing a delivery vehicle like a plasmid can elicit an enduring immune memory against the *Leishmania* parasite. Cellular response such as autophagy and apoptosis in leishmaniasis may be regulated by bistable switch to regulate BCL2 and Beclin1. Alternate expression of both genes may control homeostasis of cell to achieve parasite elimination and creating less impact on host cells (Guhe et al., 2023; Figure 2A). Hence, incorporation of a regulatory toggle switch (Soni and Singh, 2021b; Figure 2G) or bistable expression system, would necessitate administering only an inducer dose and boosters to the host to activate the immune response for the sustained development of long-term memory (Figure 2).

## Discussion

There is an urgent need to develop innovative therapeutics for leishmaniasis to overcome the limitations of current diagnosis and treatment methods. Synthetic biology approaches present novel opportunities for the creation of inventive therapeutics against leishmaniasis. For instance, researchers have utilized synthetic biology to manipulate T cells, enabling them to express chimeric antigen receptors (CARs) designed to recognize leishmanial antigens (Arya and Arora, 2021). Similarly, immune cells can be modulated using engineered synthetic circuits to detect and eliminate parasites by inducing the production of pro-inflammatory cytokines, which can stimulate NO production (Khandibharad et al., 2022). The optimization of synthetic circuit delivery is a critical aspect of advancing immunotherapy and synthetic biology applications. The choice of delivery agents and vectors plays a crucial role in the efficiency, specificity, and safety of introducing synthetic circuits into target cells. Viral vectors, Liposomes, Nanoparticles and Nanobodies may prove efficient in circuit delivery. Advances in delivery technologies continue to enhance the precision and efficacy of synthetic circuit delivery, contributing to the progress of *both in vitro* and *in vivo* applications in CL models.

The designing and construction of synthetic circuits involve principles from electrical engineering, computer science, and biology. Distinguished genetic parts and regulatory elements to build these circuits often employ iterative design and testing cycles to achieve the desired functionality. Hence the choice of design of circuit is crucial for successful modulation of immune cells. Toggle switches, Oscillators, Feedback loops and Boolean Networks are often engineered to construct synthetic circuits. Targets of synthetic circuits for functional modulation of immune cells are principally, synthetic cytokines and synthetic cytokine receptors that might control the cytokine response and enhance the enrichment of specific types of immune cells in leishmaniasis. This approach has exhibited promising outcomes in preclinical studies and holds the potential to evolve into a novel therapeutic for leishmaniasis.

Considering the high costs, issues of drug resistance, and severe side effects associated with existing treatments for leishmaniasis, there is a growing interest in leveraging synthetic circuits to achieve targeted and temporally regulated expression. Development of technologies that is suitable for synthetic circuit production in large product quantities and for producing compounds with intricate glycosylation characteristics may be a challenge (Brooks and Alper, 2021). The features of biological systems can be combined for therapeutic and diagnostic applications thanks to synthetic cells. It is an attempt to simulate a cell’s biological functions. The difficulty lies in deriving insights into the development of cell (Fu, 2013). The objective is to minimize the likelihood of resistance development against therapeutics. In our future research, we plan to develop an inducible synthetic circuit based on the tetracycline operon. This circuit will be designed to express a therapeutic peptide, and its efficacy will be assessed using macrophage cell lines, specifically RAW264.7 cells, as well as primary peritoneal macrophages. Additionally, the intention is to incorporate this synthetic circuit into a KNOCK-IN mouse model to evaluate the therapeutic effectiveness of the peptides in an *in vivo* setting. This approach aims to address the limitations of current leishmaniasis treatments and explore a more targeted and controlled therapeutic strategy.

## Conclusion

In conclusion, synthetic biology approaches have shown great promise for developing new and effective strategies to combat leishmaniasis. An effective method for synthetic circuit production is in metabolic engineering microbes to produce the synthetic circuits at large scale. The field is constantly growing because of the emergence of more affordable gene synthesis techniques, cutting-edge genome engineering tools, and the continuous development of genomic functional parts. Reprogramming circuits of this type if employed frequently in clinics in the future, synthetic circuit libraries could facilitate the seamless and cost-effective customization of treatments for individual patients. Vaccines, diagnostic tools, and novel therapeutics based on synthetic biology have shown promising results in preclinical studies and could potentially be developed into clinical candidates. However, further research is needed to optimize these approaches and ensure their safety and efficacy in humans. In the future, if the regular implementation of circuit reprogramming in clinical settings becomes commonplace, there may be a proliferation of libraries featuring diverse sensors or effectors.

## Data availability statement

The original contributions presented in the study are included in the article/supplementary material, further inquiries can be directed to the corresponding author.

## Author contributions

SK: Data curation, Formal analysis, Methodology, Resources, Software, Validation, Visualization, Writing – original draft. SS: Conceptualization, Funding acquisition, Investigation, Project administration, Resources, Software, Supervision, Visualization, Writing – review & editing.
